# Intestinal microbiota: a new force in cancer immunotherapy

**DOI:** 10.1186/s12964-020-00599-6

**Published:** 2020-06-10

**Authors:** Zhujiang Dai, Jingqiu Zhang, Qi Wu, Huiwen Fang, Chunfeng Shi, Zhen Li, Chaobiao Lin, Dong Tang, Daorong Wang

**Affiliations:** 1 Clinical Medical college, Yangzhou University, Yangzhou, Jiangsu Province China; 2 Department of General Surgery, Institute of General Surgery, Clinical Medical College, Yangzhou University, Northern Jiangsu People’s Hospital, Yangzhou, 225001 P. R. China

**Keywords:** Microbiota, Cancer immunotherapy, Checkpoint, PD-1, PD-L1, CTLA-4, ICIs, FMT

## Abstract

Cancer displays high levels of heterogeneity and mutation potential, and curing cancer remains a challenge that clinicians and researchers are eager to overcome. In recent years, the emergence of cancer immunotherapy has brought hope to many patients with cancer. Cancer immunotherapy reactivates the immune function of immune cells by blocking immune checkpoints, thereby restoring the anti-tumor activity of immune cells. However, immune-related adverse events are a common complication of checkpoint blockade, which might be caused by the physiological role of checkpoint pathways in regulating adaptive immunity and preventing autoimmunity. In this context, the intestinal microbiota has shown great potential in the immunotherapy of cancer. The intestinal microbiota not only regulates the immune function of the body, but also optimizes the therapeutic effect of immune checkpoint inhibitors, thus reducing the occurrence of complications. Therefore, manipulating the intestinal microbiota is expected to enhance the effectiveness of immune checkpoint inhibitors and reduce adverse reactions, which will lead to new breakthroughs in immunotherapy and cancer management.

Video abstract

Video abstract

## Background

Current treatments are unable to cure many cancers, mainly because of cancer’s ability to evade immune surveillance or anti-tumor disorders caused by impaired immune function [1]. In the last few years, the study of the regulation of the immune response through immune checkpoints has led to a breakthrough in therapeutic strategies in the field of oncology, bringing hope to many patients with cancer. Cancer immunotherapy reactivates the immune function of immune cells by blocking immune checkpoints (e.g., programmed death receptor 1/programmed death ligand 1 (PD-1/PD-L1), cytotoxic T lymphocyte antigen 4 (CTLA-4)) and restores the anti-tumor activity of immune cells [2, 3]. Based on considerable preclinical and clinical evidence, some immunotherapeutic drugs have been approved by the Food and Drug Administration (FDA) to treat various malignancies [4]. However, as the use of immunotherapy drugs in clinics increases, the blockade of immune checkpoints will cause an imbalance between autoimmune and immune tolerance, causing immune-related adverse events [5].

Meanwhile, sequencing technology has developed rapidly in recent years. Compared with traditional microbial culture techniques, molecular techniques using 16S rRNA or DNA / sequencing / metagenomics methods have provided more information on the microbiome and have revealed some potential immune functions of the intestinal microbiota [6, 7]. Therefore, researchers have gradually shifted their focus to study the relationship between the microbiota and cancer immunity. A growing body of evidence supports the role of microbiota in the treatment of cancer, particularly the response of the microbiota to blockade of cancer immune checkpoints [8, 9]. In the present review, we discuss the possible mechanisms of the microbiota’s effects on tumor immunotherapy and the advantages of common immunotherapies. The intestinal microbiota can improve cancer immunotherapy and patient prognosis; therefore, manipulating the microbiota will become a new force to improve cancer immunotherapy.

### Intestinal microbiota regulates immune responses in the body

The intestines are the main location of the hundreds of millions of microbes that form the microbiota. The intestinal microbiota is essential in metabolism and immunity of the host; therefore, it is considered to be an invisible organ of the human body [10]. The immune function of the microbiota starts at the intestinal level and progresses to the systemic level during an immune response.

**Fig. 1:**
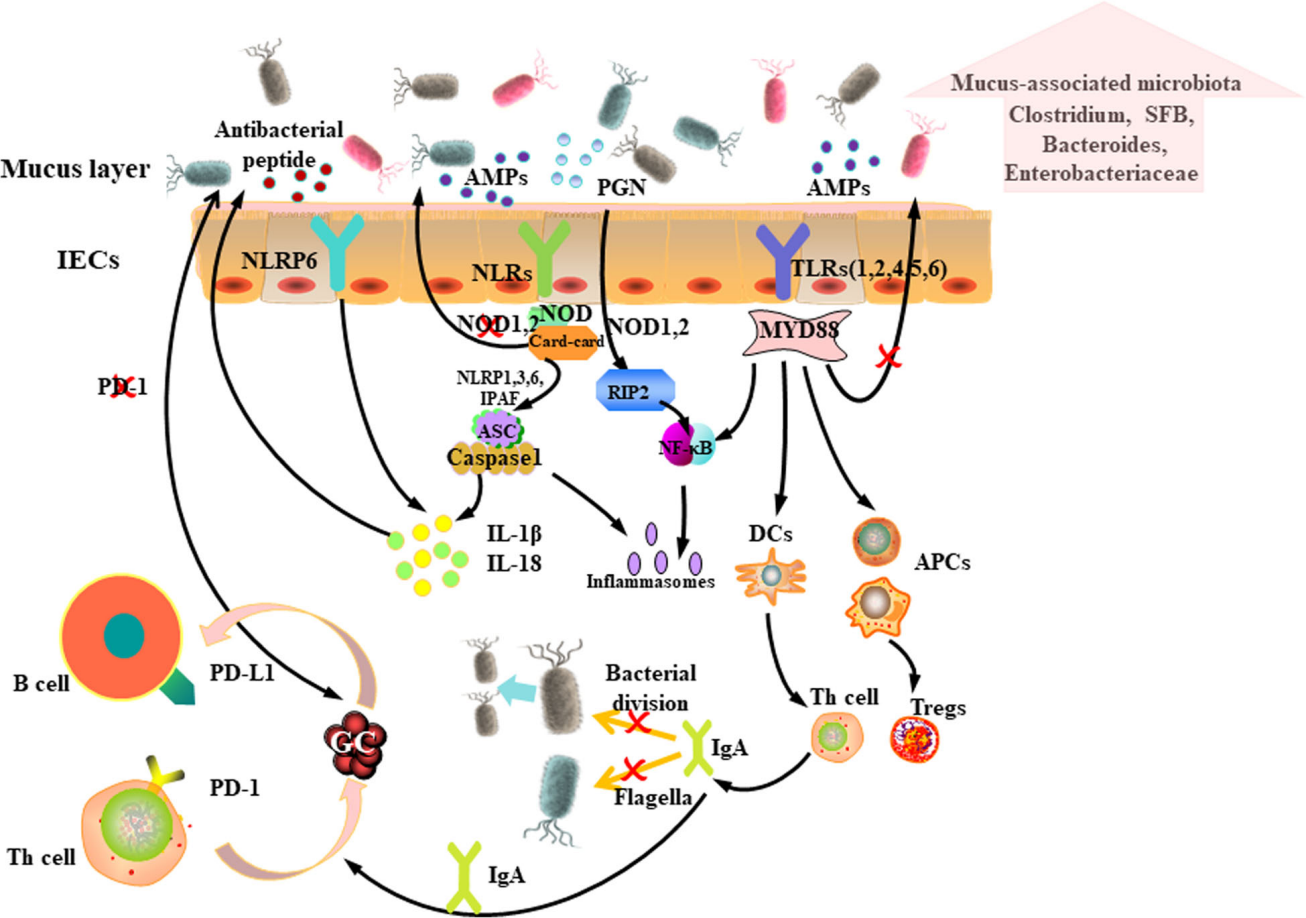
TLRs and NLRs effectively regulate intestinal immune function. The lack of the TLR adapter MYD88 will alter the composition of the microbiota, resulting in an increase in the amount of the mucus-associated microbiota. The lack of nucleoside-binding oligomeric domain protein 1 (NOD1) leads to an increase in the size of the of microbiota, including increased numbers of *Clostridium*, *Bacteroides*, segmented filamentous bacteria (SFB), and *Enterobacteriaceae*. Lack of NOD2 also leads to an increase in the size of the mucus-associated microbiota, which induces inflammation and colorectal cancer. The microbiota produce metabolites that activate NOD-, LRR-, and pyrin domain-containing 6 (NLRP6) and secretes interleukin (IL)-18, which maintains the stability of the mucus, and antimicrobial peptides. Activation of antigen-presenting cells (APCs) promotes the differentiation of CD4^+^ T cells into T helper (Th) cells and regulatory T cells (Tregs). Th cells regulate the function of the intestinal microbiota via the expression of immunoglobulin A (IgA). Furthermore, the secretion of IgA is regulated by the specific binding of PD-1 on the surface of Th cells to PD-L1 on the surface of B cells

The intestinal epithelium is a mucosal tissue, of which intestinal epithelial cells (IECs) and intraepithelial lymphocytes are the major components. Paneth cells and goblet cells are embedded between IECs, which secrete antimicrobial peptides (AMPs) and mucus, to form the first line of defense against invading pathogens. The lamina propria is located below the mucosal layer, consisting of Peyer’s plaques and immune cells [11]. Pattern recognition receptors (PRRs) are part of innate immunity and are mainly expressed in immune cells [12]. They are considered to be the bridge between innate immunity and adaptive immunity. PRRs recognize pathogen-associated molecular patterns (PAMPs) and damage-related molecular patterns (DAMPs) that affect the colonization of the intestinal microbiota. Among the more typical PRRs associated with microbial homeostasis are Toll-like receptors (TLRs) and NOD-like receptors (NLRs) [13, 14]. TLRs bind to cell membranes and effect signal transduction through myeloid differentiation of primary response protein 88 (MYD88) and TIR-domain-containing adapter-inducing interferon-β (TRIF) [15]. Most TLR signals are transmitted through MYD88, while the signals of TLR3 and some TLR4s are transmitted through TRIF [16]. TLR1, 2, and 4–6 are expressed on the cell surface and can recognize extracellular microbes, while TLR3 and TLR7–9 are thought to detect and recognize virus particles [17]. TLR2 interacts with ligands, including bacterial lipopeptides and lipoprotein acids, and forms heterodimers with TLR1 or TLR6 [15]. Then, the heterodimer binds to MYD88 and activates the nuclear factor kappa B (NF-κB) pathway under the induction of IL-1R-associated kinases 1, 2, and 4 (IRAK1, 2, and 4) [18]. However, TLR4, with lipopolysaccharide as its ligand, requires another adapter, namely the TRIF-related adaptor molecule (TRAM), to bind to TRIF [19]. The complex formed by TLR4 and TRIF then combines with MYD88 to form a common MYD88-dependent NF-κB pathway [20]. After the MYD88-NF-κB pathway is activated, pro-inflammatory factors begin to be released, initiating the inflammatory response [20]. In addition, deletion of the MYD88 signal in epithelial cells contributes to an increase in the quantity of the mucus-associated microbiota and its’ translocation to the mesenteric lymph nodes (mLNs) [21, 22] (Fig. 1). Research also showed that TLRs were strongly expressed in human colorectal cancer cells, especially TLR2 and TLR4 [23]. TLR5 is a special TLR that is thought to be related to the prevention of microbiological diseases, especially intestinal lesions caused by pathogenic adhesion of *Escherichia coli*. When TLR5 is lacking, *E. coli* flagellin cannot transmit signals through TLR5, which limits the body’s immune response [24]. TLR5-deficient mice are prone to overeating and to develop metabolic syndrome compared with wild-type mice [25]. The use of antibiotics could correct this metabolic phenotype.

NOD-like receptors (NLRs) are another class of PRRs associated with microbial disorders. The NLR family includes NODs (nucleotide-binding oligomerization domain-1), NLRPs (NACHT-, LRR- and pyrin-domain-containing proteins), IPAF (ICE-protease activating factor), NAIPs (neuronal apoptosis inhibitor factors), and class II major histocompatibility complex transactivator (CIITA) [19]. Some NLRs can identify microbial components. NOD1 and NOD2 sense the components of peptidoglycan (PGN) in the cell walls of the microbiota [15]. In this process, NOD1 is activated by γ-D-glutamyl-meso-diaminopimelic acid (iE-DAP), while NOD2 is activated by muramyl dipeptide (MDP) [26]. iE-DAP is not as widespread as MDP, only being found in the cell wall of gram-negative bacteria and a small number of gram-positive bacteria [27]. Therefore, NOD2 is always used as a general bacterial sensor to transmit inflammation signals. NODs are very active in the intestine and can recognize caspase recruitment domains (CARD-CARD) [28]. In the presence of CARD-CARD, NODs immediately form oligomers when stimulated by PGN and compete to recruit receptor interacting protein 2 (RIP2) kinase [19]. Then, the NOD1-RIP2 or NOD2-RIP2 complex further triggers the activation of transforming growth factor β-activated kinase 1 (TAK1) and NF-*κ*B, thus inducing inflammation [29]. TAK1 also phosphorylates mitogen activated protein kinase (MAPK), JUN N-terminal kinase (JNK), and extracellular activated kinase (ERK), and promotes the expression of transcription factor activated protein-1 (AP-1), which causes the release of proinflammatory mediators [30].

Other NLRs, namely NLRP1, NLRP3, NLRP6, and IPAF, are mainly involved in the assembly of inflammasomes [19]. NLRP1 can be activated by the lethal anthrax toxin [29]. NLRP3 is activated by ligands such as MDP and bacterial RNA, and forms a complex with the adapter apoptosis-associated speck-like protein containing a CARD (ASC) [23]. NLRP6 was discovered recently and is activated by the toxin released by *Listeria monocytogenes* [31]. IPAF is mainly activated by bacterial flagellin, which transmits the signal to the cytoplasm [32]. Then, NLRP1, NLRP3, NLRP6, and IPAF combine with ASC and recruit caspase-1 after forming inflammasomes in the cytoplasm, thereby promoting the release of IL-1β, thus leading to an inflammatory response [28]. All of these factors contribute to the innate immune response to the microbiota, and they have a positive effect on tissue repair and tumor monitoring on the surface of the intestinal mucosa [23].

However, in the absence of NOD1, the size of the microbiota is increased, including increased numbers of symbiotic *Clostridium*, *Bacteroides*, segmented filamentous bacteria (SFB), and *Enterobacteriaceae* [33]. Similarly, the microbial population of mice lacking NOD2 also changed, characterized by an increase in the burden of the commensal microbiota and an increase in the proportion of the mucus-associated microbiota, resulting in intestinal inflammation and colorectal cancer in mice [34]. Similar to these observations in mice, a human *NOD2* polymorphism is associated with Crohn’s disease [35]. Interestingly, the expression of NOD2 depends on the existence of the commensal microbiota, thus suggesting a negative feedback relationship between the commensal microbiota and NOD2 [36]. In addition to NOD1 and NOD2, some NLR proteins assemble into a multiprotein complex that activates caspase 1 and further releases IL-1β and IL-18 [37]. NLRP6 proteins induce intestinal epithelial inflammatory body formation. NLRP6 has been shown to be critical in maintaining intestinal microbial homeostasis [38]. Mechanistically, symbiotic microbial-derived metabolites activate NLRP6-associated inflammatory corpuscle IL-18, which maintains mucus and antibacterial peptide stability, and controls the microbial composition [39, 40].

In the adaptive immune process, antigen-presenting cells (APCs) are activated by PAMPs and then transferred into mLNs to promote the differentiation of naive T cells into CD4^+^ T cells [41]. CD4^+^ T cells differentiate into two subsets, T helper (Th) cells and regulatory T cells (Tregs). Th cells regulate the intestinal microbiota, especially microbial functions (such as flagella production) by selecting an appropriate immunoglobulin A (IgA) plasma cell bank [42]. IgA is crucial to maintain a symbiotic balance between the microbiota and the immune system. Interestingly, the most preferentially targeted microbiota for IgA is the one that proximally colonizes the mucosa and is associated with the potential pathogenicity of *E. coli* [43]. Studies on *Shigella flexneri* IgA antibodies have shown that IgA can induce the microbiota to fall into the mucous layer of the intestinal epithelium [44]. Then, IgA promotes its clearance by agglutination. IgA antibodies produced after oral inoculation with *Salmonella typhimurium* have been shown to inhibit and eliminate bacterially dividing daughter cells [45]. Although the reactivity of multi-reactive IgAs with flagellin is low, IgA might also limit bacterial movement by binding to bacterial flagellin [46]. In addition, the secretion of IgA is also regulated by the specific binding of programmed death receptor 1 (PD-1) expressed by Th cells to programmed death-ligand 1(PD-L1) on the surface of B cells [47]. IgAs produced in PD-1-deficient mice showed reduced bacterial binding capacity, leading to changes in the intestinal microbiota [48]. The changes’ main feature is that the number of *Bifidobacteria* is reduced and the number of *Enterobacteriaceae* is increased [49]. Thus, PD-1 is vital to regulate the diversity of antibodies required to maintain a full mucosal barrier. Maruya et al. also found that PD-1 affects the kinetics of B cells in the germinal center (GC) by regulating the quantity and nature of Th cells in Peyer’s patches [47]. Studies have shown that compared with wild-type mice, the frequency of clone-related sequences (with the same VH-DH-JH and ligation) in PD-1-deficient mice was reduced, resulting in impaired IgA plasma cell expansion in the GC [47]. Meanwhile, Kawamoto et al. found that the quality of IgA depends largely on the number of Th cells in Peyer’s patches. Too many Th cells lead to dysregulation of IgA precursor cells in the GC, and the defect of PD-1 can lead to an increase in Th cells [48]. When PD-1-dependent checkpoints are missing, the intestinal microbiota crosses the mucosal barrier, inducing systemic GCs to produce autoreactive antibodies [48]. Therefore, the evidence indicates that PD-1 regulates the intestinal microbiota by appropriately selecting the IgA plasma cell sequence [48]. In addition, SFB can be directly attached to IECs to stimulate the secretion of serum amyloid A [50]. These proteins belong to the acute phase response protein family and respond to inflammation. Differentiation of Th17 cells and secretion of IL-17a were achieved under the induction of serum amyloid A [50, 51]. Th17 cells are the differentiation of CD4^+^ T cells through transforming growth factor beta (TGF-β) and IL-6 and play an important role in tumors. The significant feature of Th17 cells is their ability to produce IL-17. IL-17 has six family members (IL-17a-IL-17f) [52]. Among them, the expression levels of IL-17a and IL-17f are related to tumor angiogenesis [53]. The expression of IL-17a mRNA increases with the increase of tumor invasion and pathological stage. However, IL-17a has different promotion effects on tumor angiogenesis [54]. For example, in non-small cell carcinoma, IL-17a increases vascular proliferation by promoting the angiogenic chemokines C-X-C motif chemokine ligand (CXCL)1, 5, and 8. In human melanoma blood vessels, IL-17a upregulates the expression of vascular genes through an IL-6-dependent mechanism [54]. However, IL-17f has an inhibitory effect on tumor angiogenesis [55]. In colorectal cancer, overexpression of IL-17f reduces vascular endothelial growth factor (VEGF) levels, inhibits angiogenesis, thereby playing a protective role [55]. Therefore, these two cytokines form a balance in tumor growth, which is conducive to maintaining the body’s immunity. However, there are conflicting opinions about the function of Th17 cells in tumor immunity, and that the fate of Th17 cells is regulated by many factors, including regulatory factors and intestinal bacterial antigens [56]. Among them, IL-23 can induce the expression of RUNX family transcription factor (RUNX)1 and RUNX3, which can maintain Th17 function for a long time by enhancing retinoic acid receptor-associated orphan receptor γt (RORγt) activity [57].

**Fig. 2:**
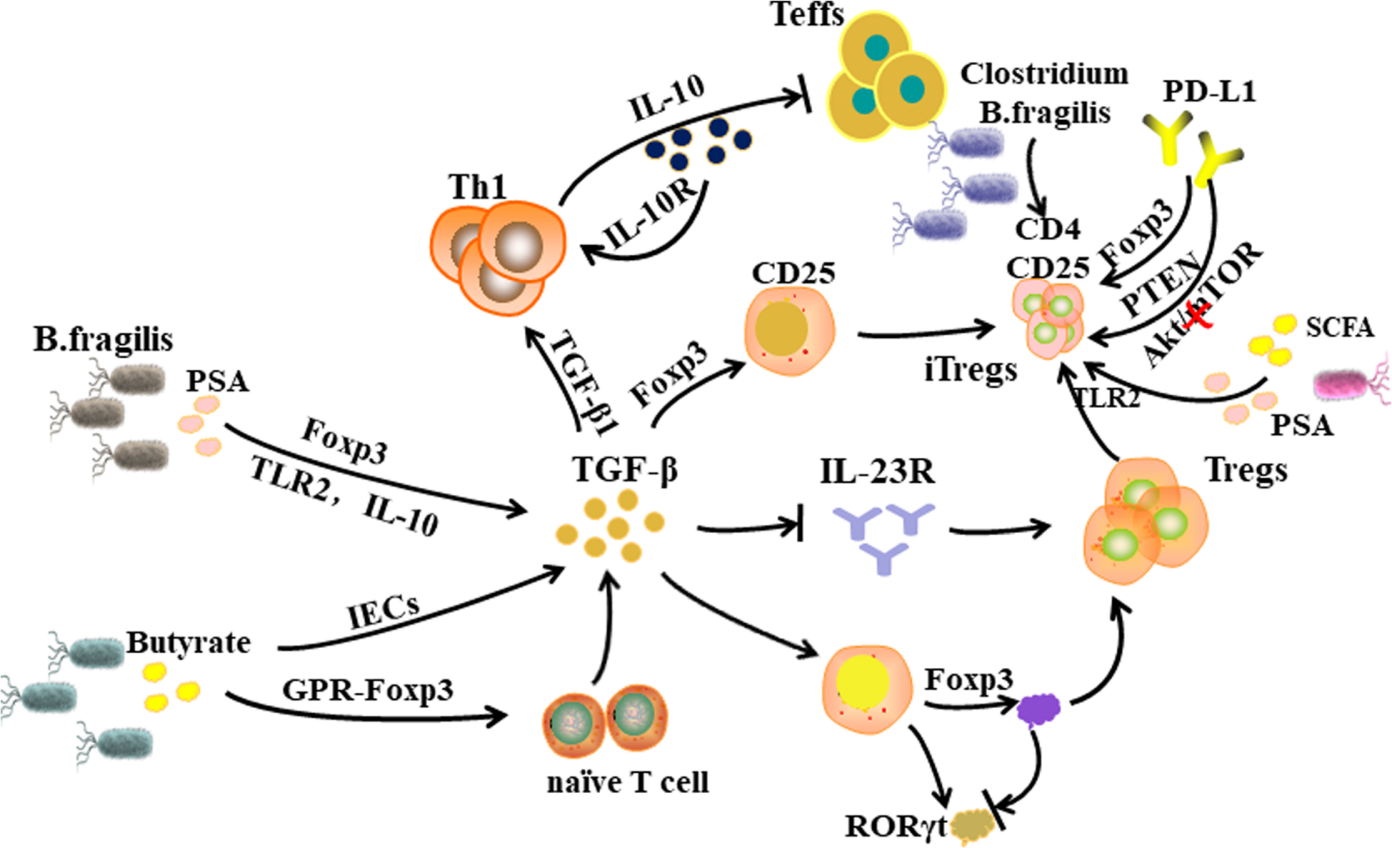
The regulation of the microbiota in adaptive immunity. *Bacteroides fragilis* stimulates TLR2 on CD4^+^ T cells by producing polysaccharide A (PSA), thereby enhancing the expression of Forkhead Box P3 (Foxp3), IL-10, and TGF-β. Butyrate activates Foxp3 via a G protein-coupled receptor (GPCR), induces differentiation of Tregs, and inhibits anti-tumor immune responses. Butyrate also indirectly promotes Treg differentiation by inducing IECs to secrete TGF-β. High concentrations of TGF-β inhibit the expression of IL-23R and promote the differentiation of Tregs. TGF-β also induces RORγt to be expressed together with Foxp3 in CD4^+^ T cells, which in turn inhibits RORγt, leading to differentiation of Tregs. Microbial metabolites SCFA and PSA can promote the proliferation of induced regulatory T cells (iTregs); however, too many iTregs infiltrating tumor tissue will weaken cancer immunity. PD-L1 can also promote the conversion of Tregs to iTregs by increasing the expression of Foxp3 and PTEN, or by inhibiting the Akt/mTOR pathway

The lamina propria of the colon is rich in Tregs, which can express PD-1 and PD-L1. Moreover, the PD-1/PD-L1 axis is thought to inhibit the response of CD4^+^ and CD8^+^ T cells [58, 59], maintaining immune tolerance to tumors and microbial antigens [60]. The inhibitory effect of Tregs on CD4^+^ T cells is mediated by cytokines such as TGF-β and IL-10 [61, 62] (Fig. 2). TGF-β1 can trigger the release of IL-10 by Th1 cells and reduce the activity of effector T cells (Teffs) [63, 64]. The microbial metabolites short chain fatty acids (SCFAs) also exhibit a regulatory effect on immune factors. SCFAs activate signal transducer and activator of transcription 3 (STAT3) and mammalian target of rapamycin (mTOR) in Th1 cells, which in turn upregulate the transcription factor B lymphocytes to induce mature protein 1 (BLIMP-1) and induce IL-10 release [65]. Meanwhile, Cottrez et al. found that IL-10 can induce feedback regulation of TGF receptor expression and enhance the response of activated T cells to TGF-β1 [66]. Forkhead box P3 (Foxp3) plays a key role in Treg development and immunosuppressive activity [67]. Mice with *Foxp3* genetic defects have dysfunctional Tregs and develop an autoimmune disease similar to lupus [67]. TGF-β induces the expression of Foxp3 in surrounding CD25 cells and converts them into CD4^+^CD25^+^ induced Tregs (iTregs) [59]. The expression of Foxp3 is controlled by the microbiota and microbial metabolites. *B. fragilis* promotes immune tolerance by producing the symbiotic factor polysaccharide A (PSA) [68]. PSA directly motivates TLR2 on CD4^+^ T cells, powerfully enhancing the expression of Foxp3, IL-10, and TGF-β [69]. Microbial-derived butyrate inhibits the activity of histone deacetylase, which activates Foxp3 via a G-protein coupled receptor (GPCR) and promotes differentiation of naive T cells into Tregs, eliminating anti-tumor immune responses [70, 71]. Furthermore, butyrate is capable of indirectly promoting the differentiation of Tregs by inducing IECs to secrete TGF-β [72, 73]. High concentrations of TGF-β prevent the expression of IL-23R and promote the differentiation of Tregs [74]. TGF-β also induces the co-expression of RORγt and Foxp3 in CD4^+^ T cells. However, in vitro, a small proportion of Foxp3 induced by TGF-β can have an inhibitory effect on RORγt, and finally lead to the differentiation of Tregs [75]. IL-6, IL-21, and IL-23 can relieve the above inhibition [76]. Inducible regulatory T cells are a subset of intestinal Tregs that develop from CD4^+^Foxp3 naive T cells, depending on microbial antigenic stimulation [77]. Research by Atarashi and Round et al. showed that an increase in symbiotic *Clostridium* species and *B. fragilis* promoted intestinal iTregs [78]. Studies also showed that symbiotic microbiota, including most Clostridia, can produce SCFAs. Among them, butyrate is involved in the generation of iTregs by inhibiting pro-inflammatory factors and inducing Foxp3 transcription [79]. *B. fragilis* strains expressing PSA can also mediate the generation of iTregs via TLR2 [79]. Francisco et al. also reported that PD-L1 induces the differentiation of iTregs by maintaining and increasing the expression of Foxp3 in iTregs [80]. PD-L1 inhibits the phosphatidylinositol-4,5-bisphosphate 3-kinase (PI3K)/protein kinase B (Akt)/mTOR signaling cascade and upregulates phosphatase and tensin homolog (PTEN) to promote the transformation of iTregs [81]. However, iTregs infiltrate tumor tissue in large quantities, inhibiting effective tumor immunity [82]. The latest research shows that iTregs might be involved in the treatment of PD-1/PD-L1 blockade, and the PD-1/PD-L1 axis might also affect the differentiation and function of iTregs [83]. However, the complex relationship between them has not been fully determined. The above-mentioned process indicates that the microbiota and their metabolites are involved in the body’s cancer immunity process.

### Tumor immunosuppression and immunotherapy

The human immune system has always been the leading force for tumor suppression. The process requires coordination of cells, tissues, and the microenvironment to maintain overall immunity. A study found that the expression of immune checkpoints such as PD-1/PD-L1 and CTLA-4 plays a pivotal role in the balance and escape phase of cancer immunity [84]. In the tumor microenvironment, activation of the PD-1/PD-L1 pathway is beneficial to tumor immune escape [2]. PD-1 is expressed in a series of activated immune cells, including T cells, B cells, natural killer (NK) cells, and dendritic cells (DCs). PD-L1/PD-L2 is mainly expressed in APCs and tumor cells [85]. When PD-L1/PD-L2 on the surface of tumor cells binds to PD-1 on the surface of T cells, T cell activation is inhibited, resulting in apoptosis of tumor-specific T cells [86]. PD-L1 is expressed in various types of cancers, especially in non-small cell lung cancer (NSCLC), melanoma, gastric cancer, liver cancer, and leukemia tumors [87]. However, unlike PD-1/PD-L1, CTLA-4 is expressed in specific Tregs and occurs in the early stages of T cell activation [88]. In contrast, PD-1 is expressed in the late phase of T cell activation [89, 90]. From the crystal structure of CTLA-4/B7, they both have higher affinity [91]. Therefore, CTLA-4 recognizes B7 ligands on the surface of tumor cells and further inhibits T cell activation [92]. A large body of evidence indicates that Tregs expressing CTLA-4 have extracellular inhibition of traditional T cells [93]. Genetic studies have also shown that Tregs’ expression of CTLA-4 is critical to control traditional T cell activation. Michella et al. also pointed out that CTLA-4 is a key effector used by Tregs to control the GC [93]. Mice lacking CTLA-4 will spontaneously develop T cell-driven lymphoproliferative syndrome, leading to early death [94].

Activation of checkpoint proteins on T lymphocytes helps tumors to escape immune surveillance. Therefore, the use of checkpoint inhibitors can reactivate the anti-cancer effects of T cells. Currently, two types of immunological checkpoint inhibitors (ICIs) that have been approved for clinical use by the FDA: Inhibitors of PD-1 or its ligand PD-L1 and inhibitors of CTLA-4 [95]. Research on ICIs has also made striking progress in determining the mechanism of checkpoints. Currently, common ICIs include anti-PD-1 (nivolumab and pembrolizumab) [96], anti-PD-L1 (durvalumab and atezolizumab) [97], and anti-CTLA-4 (ipilimumab) antibodies [98]. Many studies have shown that nivolumab in patients with solid tumors (including advanced melanoma) has good anti-tumor activity and safety [99]. Another clinical study reported the results of a randomized, double-blind, phase III trial, which showed that nivolumab improves overall survival in patients with advanced melanoma without B-Raf proto-oncogene, serine/threonine kinase (BRAF) mutations compared with dacarbazine [100]. In addition, first-line pembrolizumab monotherapy could improve overall survival and progression-free survival in patients with untreated metastatic NSCLC, with a PD-L1 tumor proportional score (TPS) of 50% or higher [101]. Although PD-1 ICIs have achieved unparalleled success among similar drugs, because of individual differences in drug resistance of patients, some patients do not gain much benefit from ICIs [102]. The goal of developing combination therapy is to help patients with cancer who benefit less from monotherapy. Hodi et al. showed that first-line nivolumab combined with ipilimumab or nivolumab alone in patients with advanced melanoma, regardless of BRAF mutation status, could obtain long-lasting, sustained clinical benefits [103]. The joint treatment was more likely to improve survival outcomes than treatment with nivolumab alone [103]. Rozeman et al. also determined the optimal dose tolerated in a trial in which ipilimumab combined with nivolumab was used to treat macroscopic melanoma (OpACIN-neo) in the naked eye (ipilimumab 1 mg/kg + nivolumab 3 mg/kg, two cycles) [104]. Some clinical trials have achieved considerable effects, and many patients are pinning their hopes on ICIs.

### The potential of intestinal microbiota in cancer immunotherapy

Unfortunately, despite patients’ expectations, a large proportion of patients with cancer are resistant to ICIs or produce only a heterogeneous, transient response [105]. Patients might have multiple complications that could prevent the safety of ICIs from being guaranteed. A meta-analysis of the use of ICIs suggested that the combination of nivolumab and ipilimumab might result in a higher risk of full-scale immune-related endocrine disease than ipilimumab or nivolumab alone [106]. Another meta-analysis acknowledged that combination therapy has a high incidence of adverse reactions and might even lead to treatment interruption [107]. Identifying more valuable immune checkpoints or increasing the sensitivity and persistence of ICIs to known checkpoints is the focus of our future research.

The human microbial community is an organ with endless potential. It not only regulates the body’s tissue metabolism, but also participates in the body’s immune regulation. It plays an important role in diseases such as gastrointestinal cancer and diabetes [18]. The intestinal microbiota has gradually emerged as a potent force in the process of cancer immunotherapy [108]. Initially, researchers were unsure whether the symbiotic microbiota affected the body’s spontaneous immune response, thus affecting the therapeutic activity of ICIs interventions. To investigate this correlation, Sivan et al. selected mice with melanoma with the same genotypes from different laboratories (From TAC and JAX), and noted the melanoma of TAC mice was more severe than that of JAX mice [109]. After feeding under the same conditions, the researchers found that tumors in TAC mice were suppressed. After feeding with a fecal suspension of two mice, it was confirmed that the commensal microbiota of JAX mice had an anti-tumor effect [110]. Finally, the researchers used JAX mouse fecal suspensions in combination with PD-L1 ICIs. Their anti-tumor effect was significantly better than that of ICIs alone, including significantly delayed tumor growth and increased tumor-specific T cell responses [109]. This experiment clarified the function of the intestinal microbiota to optimize and enhance the efficacy of ICIs, suggesting that it could be used as an adjunct to ICI treatment.

Chaput et al. also predicted the clinical response and colitis occurrence in patients with metastatic melanoma treated with ipilimumab via their baseline intestinal microbiota [111]. Based on the study data, they speculated that the ipilimumab-induced anti-cancer response and the colitis caused by the formulation might depend on the patient’s intestinal microbiota [111]. This prospective study facilitated the identification of potentially beneficial and harmful microbiota, which would allow control of the adverse risks that patients may face [111]. In fact, depending on the composition of the patient’s intestinal microbiota, PD-1 blockers (R) and non-responders (NR) could be stratified using the RECIST 1.1 standard [112]. Derosa et al. performed fecal microbial transplantation (FMT) from the feces of R patients or NR patients in sterile or antibiotic treated mice, respectively [49]. The fecal immune response of R patients to tumors was stronger than that of NR patients. Subsequently, mice immunized with feces from NR patients were supplemented with *Akkermansia muciniphila* to restore anti-cancer activity against PD-1 treatment [49, 113]. Increasing numbers of parallel studies have further confirmed the association of intestinal microbiota with ICIs [114]. However, the intestinal microbiota is rich in species, and there remains an urgent to identify a group that has specific effects on ICIs. In a study using metagenomic shotgun sequencing and unbiased metabolomic profiling to determine the efficacy of intestinal microbiota and ICIs in the treatment of patients with melanoma, researchers analyzed the patient’s stool to find the difference between the intestinal metabolites from the responder and the disease-promoting population [115]. The study found that the composition of the host intestinal microbiota was the main factor determining the response to ICIs, which was consistent with the results of preclinical mouse model studies. In addition, the results showed that sterile or antibiotic-treated mice did not respond to immunotherapy, and *Bacteroides* was required for an anti-CTLA-4 response [115]. Vetizou and colleagues also revealed that antibiotic-treated or aseptically treated mice do not respond to CTLA-4 blockade. The team revealed that T cells are involved in the specific response of *Bacteroides thetaiotaomicron* or *B. fragilis* and the efficacy of CTLA-4 blockers [116]. Further studies have found that the anti-cancer effect of CTLA-4 blockers depends on different *Bacteroides* species [116]. In that study, the *Bacteroides* were divided into three clusters, A, B, and C, based on the fecal bacteria clustering algorithm. Then, patients with melanoma were treated with ipilimumab and found to be more likely to fall into cluster C [117, 118]. Sequencing of the 16S ribosomal RNA (rRNA) gene amplicon in feces showed that the therapeutic effect of CTLA-4 ICIs was dependent on cluster C, but not clusters A and B [119]. This was mainly because the microbiota of cluster C mainly comprises immunogenic *Bacteroides*, which can restore anti-CTLA-4 monoclonal antibody (mAb) efficacy. Clusters A and B comprise tolerant *Bacteroides*, which can produce complete resistance to treatment [119, 120]. This finding indicated that patients showing resistance or no response might benefit from FMT treatment.

During the same period, Sivan discovered that the control effect of oral *Bifidobacterium* on tumors was the same as that of PD-L1 ICIs using 16S rRNA sequencing, and determined the anti-tumor effects of *Bifidobacterium*, especially *Bifidobacterium breve*, *Bifidobacterium longum*, and *Bifidobacterium adolescentis* [109, 121]. Oral administration of *Bifidobacterium* increased the infiltration of CD8^+^ T cells and enhanced the production of interferon gamma (IFN-γ). In addition, *Bifidobacterium* promoted intratumoral DC activation to improve the underlying tumor environment and anti-PD-L1 efficacy [109]. Interestingly, a recent study by Matson et al. examined fecal samples collected from patients with metastatic melanoma before immunotherapy [122]. They found that members of the microbiota such as, *Bifidobacteria longum* and *Collinsella aerofaciens* were enriched in response to anti-PD-1 immunotherapy [122]. Another study showed that *Enterococcus faecium* is abundant in the microbiota and has a synergistic effect with *Bifidobacteria* in the anti-cancer process [122]. Several other studies have confirmed that the intestinal microbiota could be a new force in anti-immunization checkpoint therapy [122, 123]. Based on the consensus of these studies, we concluded see that patients with good intestinal microbiota have an enhanced anti-tumor immune response by improving their effector T cell function in the tumor microenvironment. In contrast, patients with poor intestinal microbiota have poorer anti-tumor immune responses because of limited myeloid infiltration and reduced antigen presentation. It could be said that the microbiota controls the cancer immune setting of individuals with cancer, and it may be feasible to manipulate the intestinal ecosystem to bypass resistance to ICIs [113].

### A powerful auxiliary role of microbiota in cancer immunotherapy

It has become clear that the intestinal microbiota plays a vital role in the process of cancer immunotherapy. The intestinal microbiota mainly promotes cancer immunotherapy and optimizes the use of ICIs from the following aspects.

**Fig. 3:**
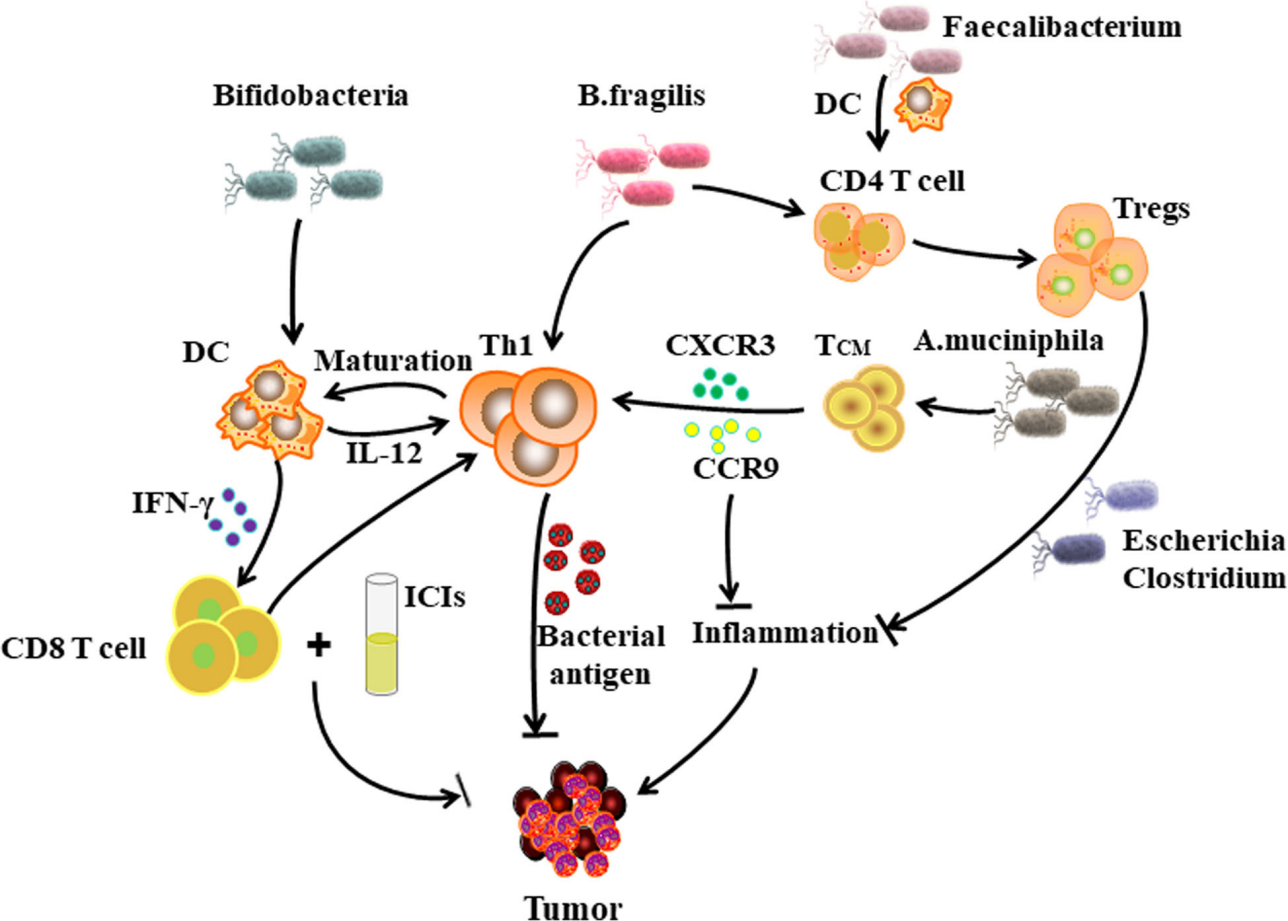
The mechanism of multiple intestinal microbiota in cancer immunotherapy. *Bifidobacteria* activates and causes DCs to secrete IFN-γ, which initiates the anti-tumor effect of CD8^+^ T cells. *B. fragilis* promotes Th1 recognition of tumor antigens and is capable of inducing DC maturation. In addition, *B. fragilis* can promote the differentiation of CD4^+^ T cells into Tregs, which further participate in anti-tumor immunity. *Faecalibacterium* induces DC maturation and promotes CD4^+^ T cell proliferation. *A. muciniphila* promotes activation of the CXCR3/CCR9 axis and participates in the migration of CD4^+^ T cells. *Escherichia* and *Clostridium* enhance the expression of CTLA-4 in Tregs, which is beneficial to tumor immunity

When PRRs such as TLRs and NODs recognize PAMPs from the microbiota, a local intestinal immune response is initiated. PAMPs promote the maturation of DCs through interaction with PRRs. *Bifidobacteria* are capable of inducing transcription of DC genes and promoting their maturation [109]. This process facilitates the recruitment and activation of lymphocytes and enhances the efficiency of antigen presentation. In addition, the threshold for the activation of DCs by *Bifidobacteria* is downregulated, meaning that the concentration of antigen required to initiate T cells is reduced, and sensitivity is increased. Studies have shown that at lower antigen concentrations, DCs upregulate IFN-γ levels, initiate the proliferation of tumor-specific CD8^+^ T cells, and produce synergistic anti-tumor effects with ICIs [124, 125] (Table 1). This process promotes activation of DCs in the spleen and tumors, improving basal tumor control and anti-PD-L1 efficacy (Fig. 3). APCs can also detect the intestinal microbiota without microbiota translocation [126, 127]. The microbiota-mediated immune response not only activates an inflammatory response in the mucosa, but also induces the differentiation of pathogenic Th17 (pTh17) and Th1 cells in the secondary immune organs [128].

**Table 1:** Regulation of intestinal microbiota in cancer immunotherapy

Microbiota (or products)	Immune regulation	Impact on cancer immunotherapy
Bifidobacteria	Promoting maturation of DCs Activating lymphocytes Upregulating IFN-γ and increasing pro-inflammatory cytokine Initiating the proliferation of tumor-specific CD8^+^ T cells	Enhancing PD-1 blockade
*B. fragilis*	Activating Th1 cells Promoting Tregs proliferation Promoting maturation of DCs	Enhancing CTLA-4 blockade
A. muciniphila	Increasing CXCR3^+^CCR9^+^CD4^+^ T cells	Enhancing PD-1 blockade
Escherichia Clostridium	Inducing the differentiation of Tregs and inhibiting inflammation	Enhancing CTLA-4 blockade
Faecalibacterium	Promoting the proliferation of CD4^+^ or CD8^+^ T cells Promoting the production of Tregs and upregulating the expression of ICOS	Enhancing PD-1 blockade Enhancing CTLA-4 blockade
Bacteroides	Upregulating the system’s MDSC and Tregs Causing a systemic inflammatory response through the TLR-NF inflammatory pathway	Impeding PD-1 blockade Impeding CTLA-4 blockade
microbial-derived SCFAs	Promoting the differentiation of Tregs	Enhancing CTLA-4 blockade

*B. fragilis* activates Th1 cells to cross-react with bacterial antigens and new tumor antigens, enhancing the efficacy of anti-CTLA-4 [129]. At the same time, colonization of germ free mice with *B. fragilis* and *B. cepacia* reduced the toxicity induced by anti-CTLA-4 mAb [116]. This might be related to the ability of *B. fragilis* to promote Treg proliferation. Monoclonalization by *B. fragilis* and *Bifidobacteria* also promotes the conversion of CD4^+^ T cells to Tregs [130]. When the IL-10 signal is blocked, the disturbance of the intestinal mucosal barrier is further increased. This might be caused by intestinal toxicity caused by pTh17 cells, such as colitis [131]. It is worth noting that the efficacy of oral *B. fragilis* is associated with Th1 immune responses induced in lymph nodes and DC maturation in the tumor bed [132]. Among them, plasma cell-like DCs (pDCs) activate lamina propria DCs, which can promote the absorption of *B. fragilis* [133]. Alternatively, absorption of soluble bacterial products by DCs promotes DC maturation and the production of IL-12, which in turn allows for the initiation of T cells (such as Th1) [134]. IL-12 might be produced as a result of *B. fragilis* mobilizing the lamina propria of CD11b^+^ DC [135]. These processes might be involved in the anti-tumor immune responses by T cells homologous to tumor antigens or cross-reactive bacterial antigens.

Colonization by the acidophilic *Akkermansia muciniphila* and *Enterococcus hirae* is associated with the appearance of CD4^+^ central memory T cells (T_CMs_) in tumors and the coding of mLNs [113]. C-X-C motif chemokine receptor 3 (CXCR3)/ C-C motif chemokine receptor 9 (CCR9) are chemokine receptors expressed by T_CMs_ [136]. The CXCR3 and CCR9/CCL25 axes are associated with progression free survival (PFS) and overall survival (OS) prolongation in some patients with advanced cancer [137]. CXCR3 is also involved in enlisting Th1 cells to inflammatory lesions. The CCR9/CCL25 axis is involved in T cell chemotactic migration, and Th cells expressing CCR9 exhibit site specificity during inflammation [138, 139]. Some data suggest that T cell epitopes are shared between the microbiota and tumor cells [113]. Under this model, T cells might exert anti-tumor effects through CD4^+^ T cell CD8^+^ T cells in response to cross-reactivity of bacterial antigens [140]. Balachandran et al. found that T cell clones around and within tumors are specific for both new antigens and predict their cross-reactivity with microbial epitopes [141]. Therefore, microbiota has a certain promotion effect on the blocking effect of PD-1.

In addition, immune cell detection showed that intestinal *Faecalibacterium* increased the role of DC and other APCs to promote the proliferation of CD4^+^ or CD8^+^ T cells, which is conducive to enhancing the blocking effect of PD-1 [142]. Furthermore, *Faecalibacterium* enhances the blocking effect of CTLA-4 by promoting the production of Tregs and upregulating the expression of inducible T cell costimulatory (ICOS) [143]. However, some members of the microbiota have antagonistic effects on ICIs. *Bacteroides* blocked PD-1 blockade by upregulating the system’s myeloid-derived suppressor cells (MDSCs) and Tregs [144]. *Bacteroides* can also cause a systemic inflammatory response through the TLR-NF inflammatory pathway, hindering the blocking effect of CTLA-4 [145].

The intestinal microbiota and its metabolites are beneficial to activate Tregs [146]. SCFAs are microbial metabolites that affect many characteristics of host immunity [147]. Intestinal microbial-derived SCFAs, such as butyrate and propionate, promote the differentiation of Tregs and increase the size of the Tregs pool by increasing the acetylation level of histone H3 in the *Foxp3* promoter region and the conserved non-coding region [148–150]. The high expression of CTLA-4 in Tregs means that the status of Tregs at baseline is critical to determine CTLA-4 blockade [151]. Some members of the microbiota, such as *Escherichia* and *Clostridium*, can induce differentiation of Tregs and inhibit the occurrence of inflammation. It is speculated that anti-inflammatory microbiota and SCFAs induce proliferation and differentiation of Tregs, resulting in higher levels of CTLA-4 [150]. Although elevated CTLA-4 levels are beneficial for tumors to evade immune surveillance, they can increase sensitivity to CTLA-4 blockade by relieving immunosuppression of the gut and tumor tissues [152]. The involvement of Tregs is more pronounced in the blocking of CTLA-4 than in PD-1 blockade. Therefore, theoretically, patients receiving CTLA-4 blockade are more likely to benefit from enhanced T cells.

These underlying mechanisms might contribute to microbial mediation of anti-tumor immune regulation in the context of intestinal inflammation, such as chemotherapy drugs that cause mucositis, or anti-CTLA-4 treatment [153, 154]. Thus, the intestinal microbiota has been recognized as a major force in the process of cancer immunity.

### The future of the intestinal microbiota in cancer immunotherapy

**Fig. 4:**
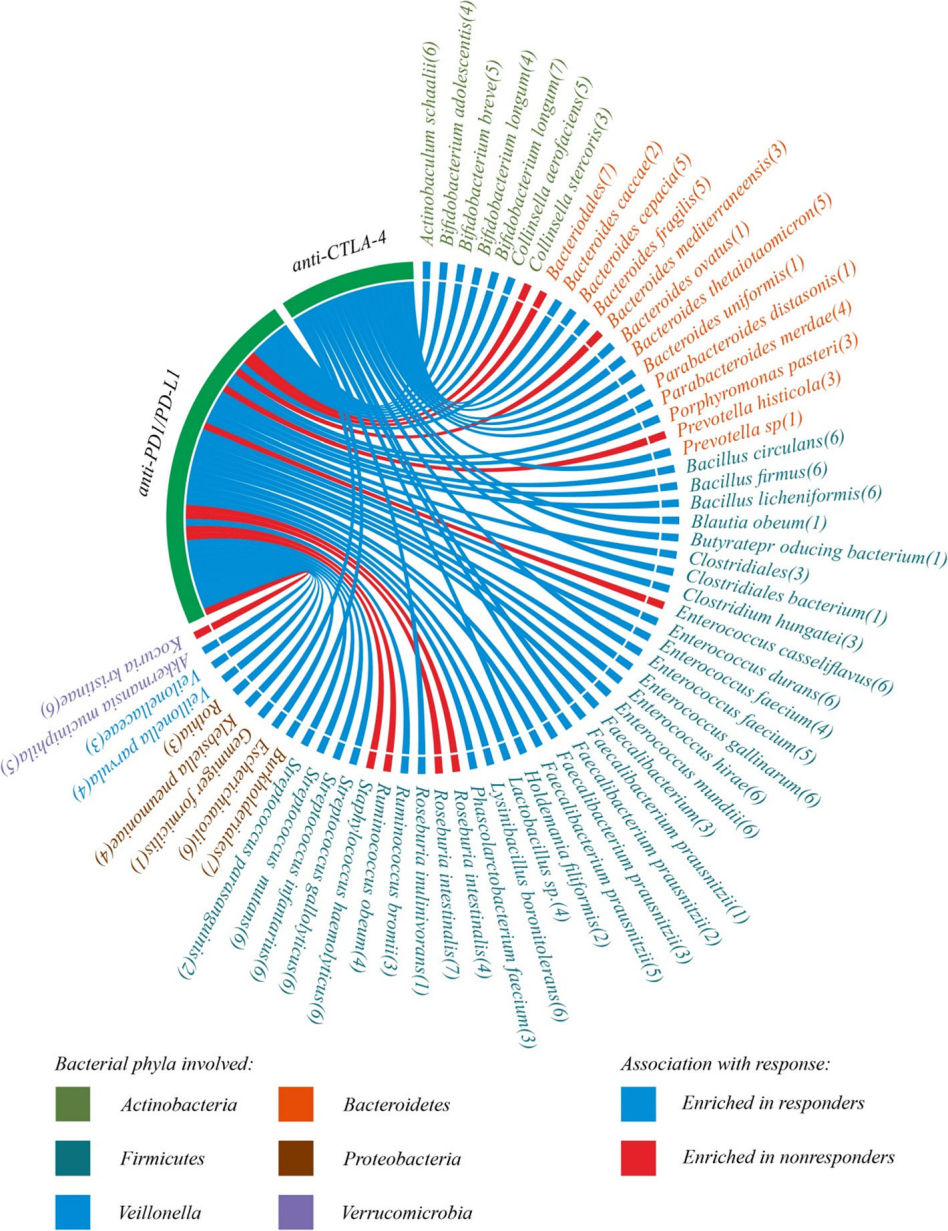
Differences in microbial enrichment during immunotherapy. The Circos diagram illustrates the different effects of different members of the microbiota in the treatment of ICIs. Blue bands represent members of the microbiota that are enriched during the treatment of effective ICIs. Red bands represent members of the microbiota that are enriched during the treatment of ineffective ICIs. The numbers in parentheses are the source of the reference. (1) Chaput et al. [111] (2) Frankel et al. [115] (3) Gopalakrishnan et al. [123] (4) Matson et al. [122] (5) Routy et al. [155] (6) Routy et al. [113]. (7) Temraz et al. [156]

As a result of ongoing research, we predict that the intestinal microbiota will gradually occupy an increasingly prominent position in cancer immunotherapy. Currently, the mechanism of the effects of the intestinal microbiota in cancer immunotherapy is not well understood; however, some ongoing clinical trials will help to reveal the potential of the intestinal microbiota in tumor development and cancer immunotherapy. We have summarized the clinical trials investigating the intestinal microbiota involvement in cancer immunotherapy in recent years (Table 2). Meanwhile, we also compiled a schematic diagram showing the enrichment of the intestinal microbiota in the process of cancer immunotherapy (Fig. 4). This evidence will provide a good reference for the effectiveness of the intestinal microbiota in the immunotherapy process.

**Table 2:** FDA-approved trials of microbial-related immunotherapy

NCT Number	Title	Status	Conditions	Interventions	Phases
NCT02960282	Gut Microbiome in Fecal Samples From Patients With Metastatic Cancer Undergoing Chemotherapy or Immunotherapy	Recruiting	Metastatic Carcinoma Stage IV/IVA/IVB Colorectal Cancer	Procedure: Biospecimen Collection Other: Laboratory Biomarker Analysis	
NCT03341143	Fecal Microbiota Transplant (FMT) in Melanoma Patients	Recruiting	Melanoma	FMT with Pembrolizumab	Phase 2
NCT03353402	Fecal Microbiota Transplantation (FMT) in Metastatic Melanoma Patients Who Failed Immunotherapy	Recruiting	Melanoma Stage IV Unresectable Stage III Melanoma	FMT	Phase 1
NCT03370861	How Microbes and Metabolism May Predict Skin Cancer Immunotherapy Outcomes	Recruiting	Skin Cancer|Melanoma Merkel Cell Carcinoma Squamous Cell Carcinoma of the Skin Basal Cell Carcinoma	Immunotherapy	
NCT03383107	Effect of Radiotherapy Variables on Circulating Effectors of Immune Response and Local Microbiome	Recruiting	Breast Cancer|Prostate Cancer		
NCT03557749	Monitoring of Immune and Microbial Reconstitution in (HCT) and Novel Immunotherapies	Recruiting	Immune and Microbial Reconstitution Recurrent Malignant Cell Therapy/Immunotherapy Patients	Diagnostic Test: Blood Sample/Stool Sample Gastrointestinal biopsy × 2–4/ Apheresis Product/Final cellular product	
NCT03595683	Pembrolizumab and EDP1503 in Advanced Melanoma	Recruiting	Melanoma (Skin)|Melanoma	Pembrolizumab Biological: EDP1503	Phase 2
NCT03643289	Predicting Response to Immunotherapy for Melanoma With Gut Microbiome and Metabolomics	Recruiting	Melanoma (Skin)		
NCT03686202	Feasibility Study of Microbial Ecosystem Therapeutics (MET-4) to Evaluate Effects of Fecal Microbiome in Patients on Immunotherapy	Recruiting	All Solid Tumors	Biological: MET-4	Early Phase 1
NCT03772899	Fecal Microbial Transplantation in Combination With Immunotherapy in Melanoma Patients (MIMic)	Recruiting	Melanoma	FMT	Phase 1
NCT03797170	Design of New Personalized Therapeutic Approaches for Diffuse Large B-cell Lymphoma	Recruiting	Diffuse Large B Cell Lymphoma	Gut microbiota samples	
NCT03817125	Melanoma Checkpoint and Gut Microbiome Alteration With Microbiome Intervention	Recruiting	Metastatic Melanoma	Placebo for antibiotic Vancomycin pretreatment Nivolumab/SER-401/SER-401	Phase 1
NCT03891979	Gut Microbiome Modulation to Enable Efficacy of Checkpoint-based Immunotherapy in Pancreatic Adenocarcinoma	Not yet recruiting	Pancreatic Cancer	Antibiotics and Pembrolizumab	Phase 4

The development of these clinical trials will remove obstacles for the use of the intestinal microbiota to optimize and assist immunotherapy using ICIs. First, the intestinal microbiota can reduce complications during cancer immunotherapy. Currently, there are some complications associated with the use of ICIs. The most common toxic response when using ICIs is associated colitis. The cause of the disease is obscure. Interestingly, the lactic acid bacteria *Lactobacillus reuteri* can completely eliminate ICIs-associated colitis, and improve weight loss and inflammation [157]. The protective effect of *L. reuteri* might be related to the decrease in lymphocyte distribution [158]. Second, the intestinal microbiota enhances the nutritional absorption capacity of patients with cancer and enhances their anti-tumor ability. The emergence of tumor micro-ecological immune nutrition has further paved the way for the development of the microbiota as tool in cancer immunotherapy [159]. The main function of the intestinal microbiota is to help the host to digest and metabolize food [160]. However, in patients with cancer, their intestinal function is often destroyed, and they find it difficult to utilize the nutrition in the diet. The addition of micro-ecological preparations to parenteral nutrition could effectively interfere with the environment of intestinal disorders, re-establish a good tumor microenvironment, and play a role in anti-tumor immunity [161]. Third, microbiota research is expected to lead to the design of a vaccine against tumors. A recent microbial-based cancer vaccine has shown its utility [162]. This cancer vaccine prevents the growth of squamous cell carcinoma expressing epidermal growth factor receptor (EGFR) vIII and induces EGFR vIII-specific cellular immunity [162]. This work is exciting for the study of anti-tumor immunity, and represents a new breakthrough in the design of the microbiota. Fourth, FMT is expected to be the most direct biooptimization tool for cancer immunotherapy. FMT is a popular and significant technology that has been used clinically to treat recurrent *Clostridium difficile* infections [163].

As microbiota research shifts from correlation studies to mechanistic studies, the activity of specific microorganisms and their products will be validated in areas such as inflammatory bowel disease and cancer. Although FMT is still in its infancy in clinical trials of cancer, it still represents a milestone in cancer therapy research. For example, clinical trial NCT03353402 proposes to change the intestinal microbiota of patients with melanoma who have failed immunotherapy using FMT. Another clinical trial (NCT03341143) is investigating the feasibility of FMT in patients with melanoma who are resistant to PD-1 ICIs. Furthermore, safety studies using FMT in combination with immunotherapy (pembrolizumab or nivolumab) are also being tested in a clinical trial (NCT03772899). These clinical trials will further examine the position of FMT in cancer immunotherapy.

## Conclusion

The intestinal microbiota has shown its potential as a major force in cancer immunotherapy. It not only participates in regulating the body’s immunity, but also assists in optimizing the therapeutic effects of ICIs. At the same time, as a tool for adjuvant therapy and the comprehensive evaluation of patients’ benefits, the targeted benefits of microbiota will gradually become clear, which could be considered in combination with FMT. Although the mechanisms of the effects of the microbiota are unclear, emerging technologies such as microbiome-wide association study (MWAS) and 16S rRNA sequencing will provide clarity in the near future. Not only do we need to fully understand how the microbiota regulates cancer immunotherapy in the context of preclinical models and clinical trials, but more importantly, we need to use these data to develop immunotherapeutic probiotics to help improve the efficacy of immunotherapy in patients.

## Data Availability

Not applicable.

## Abbreviations

AMPs: Antimicrobial peptides
APCs: Antigen-presenting cells
Blimp-1: B lymphocytes to induce mature protein 1
CTLA-4: Cytotoxic T lymphocyte antigen 4
EGFR: Epidermal growth factor receptor
DAMPs: Damage-related molecular patterns
FMT: Fecal microbial transplantation
GPCR: G-protein coupled receptor
ICIs: Immunological checkpoint inhibitors
IECs: Intestinal epithelial cells
mLNs: Mesenteric lymph nodes
MYD88: Myeloid differentiation of primary response protein 88
NOD1: Nucleoside-binding oligomeric domain protein 1
NLRs: NOD-like receptors
NLRP6: NOD-, LRR-, and pyrin domain-containing 6
NSCLC: Non-small cell lung cancer
pTh17: Pathogenic Th17
PD-1/PD-L1: Programmed death receptor 1/programmed death-ligand 1
PRRs: Pattern recognition receptors
PAMPs: Pathogen-associated molecular patterns
PTEN: Phosphatase and tensin homolog
RORγt: Receptor-associated orphan receptor γt
TPS: Tumor proportional score
TLRs: Toll-like receptors
